# Nonstationary signal extraction based on BatOMP sparse decomposition technique

**DOI:** 10.1038/s41598-021-97431-z

**Published:** 2021-09-09

**Authors:** Shuang-chao Ge, Shida Zhou

**Affiliations:** School of Instruments and Electronics, North University of China, Taiyuan, 030051 China

**Keywords:** Computational science, Pure mathematics

## Abstract

Sparse decomposition technique is a new method for nonstationary signal extraction in a noise background. To solve the problem of accuracy and efficiency exclusive in sparse decomposition, the bat algorithm combined with Orthogonal Matching Pursuits (BatOMP) was proposed to improve sparse decomposition, which can realize adaptive recognition and extraction of nonstationary signal containing random noise. Two general atoms were designed for typical signals, and dictionary training method based on correlation detection and Hilbert transform was developed. The sparse decomposition was turned into an optimizing problem by introducing bat algorithm with optimized fitness function. By contrast with several relevant methods, it was indicated that BatOMP can improve convergence speed and extraction accuracy efficiently as well as decrease the hardware requirement, which is cost effective and helps broadening the applications.

## Introduction

### Background information

Signal extraction and signal–noise separation are always two of the research focuses in modern signal processing^1^, which are commonly used in biomedical signal features extraction, vibration signal analysis, seismic signal detection^2^, sound signals recognition^3^. In practical applications, such signals are often submerged in a variety of vibration or electromagnetic noise, and the occurrence times of the target signals are random, which are typical nonstationary signals. Fourier transform is one of the most classic signal analysis and extraction method, but it cannot accurately describe nonstationary signals^4^. In recent years, new theories and technologies continue to appear in signal extraction. For example, wavelet decomposition (WD)^5,6^, empirical mode decomposition^7^, Hilbert–Huang Transform (HHT), variational mode decomposition (VMD) algorithm^8^. These methods need to meet certain conditions to work, for example the decomposition levels, modal number, and termination thresholds.

To achieve a more flexible, concise and adaptive signal decomposition, researchers proposed sparse decomposition. This method represents the signal with as few atoms as possible in a given redundant dictionary by matching pursuit (MP) algorithm^9^, which is a greedy algorithm for sparse decomposition. Various new evaluation criteria and basis pursuit, orthogonal matching pursuit algorithm (OMP)^10^, and time–frequency spectrum segmentation methods^11^ were generated to select a set of optimal atoms from the constructed over-complete dictionary. In principle, if the dictionary redundancy is high enough and the iterations is large enough, the target signal can be perfectly extracted by OMP. On this basis, some general improved algorithms were proposed for example Regularized Orthogonal MP (ROMP)^12^ and Compressive Sampling MP (CoSaMP)^13^. These methods require the signal Sparsity *K* for efficient execution, but *K* is generally unknown in practice. Sparsity Adaptive MP (SAMP) was proposed for signal reconstruction without prior information of the sparsity, but it is more complex than other greedy algorithms under large sparsity level^14^. And improper initial step size will lead to excessive decomposition for SAMP. The accuracy of signal sparse decomposition mainly depends on the redundancy and refinement accuracy of the redundant dictionary. Over or under estimation as well as long-time running will appear in these algorithms under the condition of large sparsity. Generally, the greater the redundancy and refinement, the greater the probability of accurate signal decomposition. However, for the greedy algorithm mentioned above, these are at the cost of algorithm efficiency. The accuracy and efficiency are exclusive.

Aiming at two main research hotspots including sparse decomposition algorithm and over-complete atom dictionary of signal sparse decomposition^15^, we designed two typical universal atoms, and proposed an adaptive feature-based atom construction method for the extraction of non-stationary signals with unknown sparsity. Redundant dictionary is obtained by extending the feature-based atoms, which can balance the completeness and redundancy. A signal matching tracking extraction algorithm was developed based on the bat algorithm and OMP, which successfully combined the accuracy and efficiency and could effectively realize nonstationary time domain signal extraction.

### Classical signal sparse decomposition algorithms

Signal sparse decomposition represents a signal by specific combinations of some atoms in a dictionary. For a given dictionary, the optimal combination can be accurately determined when all possible combinations were calculated. However, exhausting all combinations in a dictionary is a non-deterministic polynomial problem that is almost impossible to achieve for large dictionary bases. So, the requirement was changed to finding a suboptimal combination from the dictionary with the lowest possible number of atoms and the smallest possible extraction error. This will reduce the computational complexity significantly, and the MP algorithm is one of the algorithms that can achieve this requirement.

Assume that the represented signal is *x* with length of *N*. Let *ℝ* denote the Hilbert space in which a dictionary matrix *D* composed of a set of vectors {*g*_1_*, g*_2_*,…,g*_*n*_}. Each vector is an atom with the same length *N* and these vectors have been treated as normalized as $\left\| g \right\|_{2}^{{}} = 1$.

With *ξ*_*1*_ = *x*, the MP algorithm selects one atom at a time from the dictionary matrix *D* that best matches *x*, satisfying (1),

1 $$ c_{i} = \left| {\left\langle {x \cdot g_{{i_{best} }} } \right\rangle } \right| = \max_{{i \in \left( {1, \ldots n} \right)}} \left| {\left\langle {x \cdot g_{i} } \right\rangle } \right|, $$

where *i*_*best*_ is the index of the best matching atom in *D*. $\left\langle \cdot \right\rangle$ is the inner product function.

The signal* x* is then decomposed into two parts*, a* sparse approximation $$\overset{\lower0.5em\hbox{$\smash{\scriptscriptstyle\frown}$}}{x}$$ and an approximation residual *ξ*_2_:

2 $$ x = \overset{\lower0.5em\hbox{$\smash{\scriptscriptstyle\frown}$}}{x} + \xi_{2} = \left\langle {x \cdot g_{{i_{best} }} } \right\rangle g_{{i_{best} }} + \xi_{2} . $$

Continues to select the atoms that best matches *ξ*_2_, iterating repeatedly and eventually the signal *x* can be approximated as a linear sum of these atoms:

3 $$  \overset{\lower0.5em\hbox{$\smash{\scriptscriptstyle\frown}$}}{x}  = \sum\limits_{{i = 0}}^{{n - 1}} {\left\langle {\xi _{i}  \cdot {g_{{i_{{best}}}}} } \right\rangle {g_{{i} _{{{{best}}}} }} ,} \;x -  = \xi _{n}. $$

**Figure 1:**
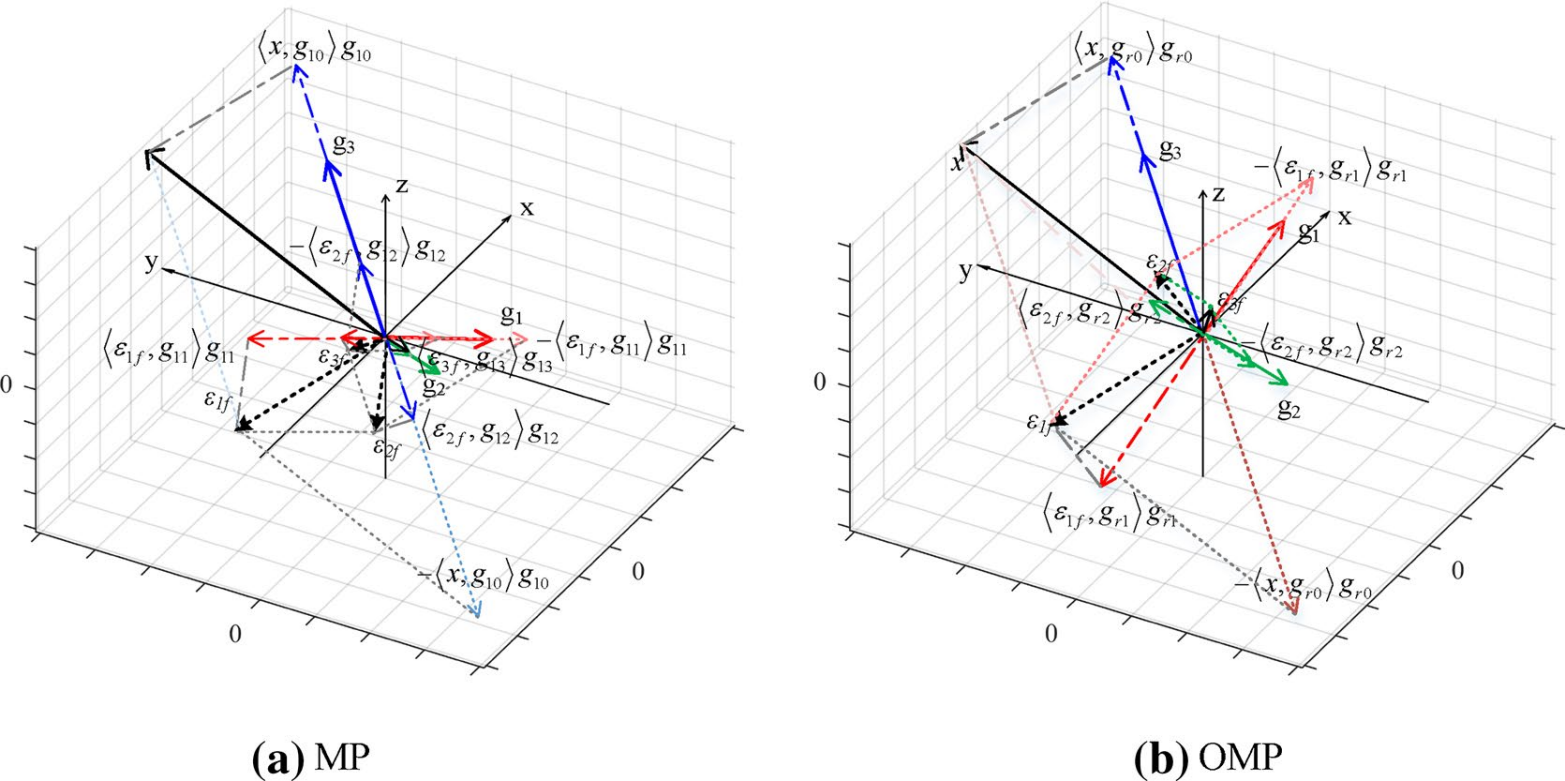
Convergence process of MP and OMP.

For MP algorithm, the non-orthogonality between the vertical projection of the signal (or residuals) on the selected atoms and the residuals will lead to suboptimal iterative results instead of the best optimal, and convergence requires many iterations. The OMP algorithm is the orthogonalization of all selected atoms at each step of the decomposition, which makes the convergence faster with the same accuracy requirement. The convergence process of MP and OMP are described by a dictionary *D* with length of three, as shown in Fig. 1. However, although the OMP algorithm reduced iterations to some extent, it had to calculate the current residual and the inner product of all atoms within the current dictionary during each iteration, resulting in unsatisfied effectiveness. Therefore, this paper introduced the bat algorithm (BA) to optimize the matching tracking algorithm.

### Bat algorithm presentation

The basic flow of bat algorithm is as follows:

1. Initialization: the best fitness *Fit*_*best*_, bat population number *N*_*pop*,_, the max bat generation *N*_*gen*_,, the current generation *n* = 0, initial flight frequency *f*_*0*_ = {*f*_i_^0^|*i* = 1,2,…, *N*_*pop*_}, acoustic loudness *A*^*0*^ = {*A*_i_^0^|*i* = 1,2,…, *N*_*pop*_} and pulse emission frequency *r*_*0*_ = {*r*_i_^0^|*i* = 1,2,…, *N*_*pop*_}. The initial location of the bat colony is randomly generated according to (4):
  4 $$ P^{0} { = }\left\{ {P_{i}^{0} \left| {i = 1,2,...,N_{pop} } \right.} \right\},\quad P_{i}^{0} \in \left( {P_{\min } \,P_{\max } } \right), $$
2. The best position *P*^*n*^_*best*_ is determined by the fitness function *Fit*^*n*^.
  5 $$ Fit^{n} \left( {P_{best}^{n} } \right){\text{ = arg }}\mathop {{\text{min}}}\limits_{{N_{pop} }} \left( {Fit^{n} } \right). $$
3. Update the velocity and position of the individual bat:
  6 $$ \left\{ \begin{array}{*{20}l} f_{i} = f_{\min } + r_{1} \times \left( {f_{\max } - f_{\min } } \right)  \\ v_{i}^{n} = v_{i}^{n - 1} + \left( {P_{best} - P_{i}^{n - 1} } \right) \cdot f_{i} \\ P_{i}^{n} = P_{i}^{n - 1} + v_{i}^{n} \\ \end{array} \right., $$
  where *r*_*1*_ was a random number, satisfying *r*_*1*_∈[0,1]; *f*_*i*_ was the search pulse frequency of the *i*-th bat; *v*_*i*_^*n*^ denoted the velocity of the *i*-th bat in the *n-th* igeneration, *P*_*i*_^*n*^ denoted the position of the *i*-th bat in the *n-*th igeneration; and *P*^*n*^_*best*_ is the current global optimal solution.
4. Generate a random number *r*_2*i*_ ∈ [0,1] for each bat and update bat position according to (7).
  7 $$ \left\{ \begin{array}{*{20}l} global optimization:P_{i}^{n + 1} { = }P_{i}^{n} + v_{i}^{n + 1} ,r_{2i}^{{}} \le r_{i}^{n} \\ local optimization:P_{i}^{n + 1} { = }P_{i}^{n} + \lambda_{ri} \mathop {A^{n} }\limits^{\_\_} * \left( {P_{\max }^{{}} - P_{\min }^{{}} } \right),r_{2i}^{{}} > r_{i}^{n} \\ \end{array} \right., $$
  where: *η* was a random number, satisfying *η* ∈ [− 1; 1] and *Ā*^*n*^ was the mean fitness of the bat population.
5. Update the fitness
  8 $$ Fit^{n + 1} \left( i \right){ = }Fit\left( {P_{i}^{n + 1} } \right). $$
6. For each bat, a random number *r*_3*i*_ is generated, and update the position:
  9 $$ \left\{ {\begin{array}{*{20}l} {P_{i}^{n + 1} { = }P_{i}^{n + 1} ,} & {r_{3i} > A_{i}^{n} \& \& Fit_{i}^{n + 1} < Fit_{i}^{n} } \\ {P_{i}^{n + 1} = P_{i}^{n} ,} & {Fit_{i}^{n + 1} = Fit_{i}^{n} ,\;otherwise} \\ \end{array} } \right.. $$
7. The fitness and pulse emission frequency are updated:
  10 $$ \left\{ \begin{array}{*{20}l} r_{i}^{n + 1} { = }r_{i}^{0} \left( {1 - e^{ - \gamma n} } \right)  \\ A_{i}^{n + 1} = \lambda A_{i}^{n}  \\ \end{array} \right.. $$
  where, *λ* ∈ (0,1), *γ* > 0, when $$n \to \infty$$, $$A_{i}^{n} \to 0$$, $$r_{i}^{n} \to r_{0}$$.
8. Find the current matching atom based on the optimal solution.
9. The random perturbation of the current optimal solution in step 4 can effectively avoid the iterative result from falling into a local optimal solution, which helped to find the global optimal solution fast and accurate.
10. The Ackley function iss used to test the BA. The expression of the Ackley function is as follows:
  11 $$ f(x) = - c_{1} \exp \left( { - 0.2\sqrt {\frac{1}{n}\sum\limits_{j = 1}^{n} {x_{j}^{2} } } } \right) - \exp \left( {\frac{1}{n}\sum\limits_{j = 1}^{n} {\cos \left( {2\pi x} \right)} } \right) + e. $$

**Figure 2:**
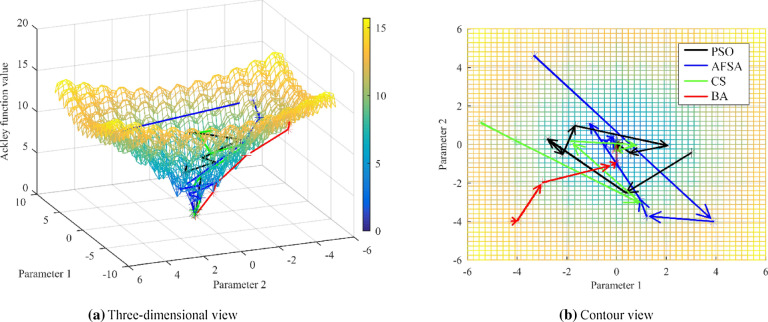
The optimal trajectory of different methods. (**a,b**) Show the 3D view and contour attempt of the optimal trajectory, respectively. Colors: black: the merit-seeking trajectories of PSO, blue: the merit-seeking trajectories of AFSA, green: the merit-seeking trajectories of CS, red: the merit-seeking trajectories of BA.

In this study, *n* = 2, *c*_*1*_ = 20, *e* = 2.71289. the Ackley function was taken as the fitness function and the global minimum of this function was searched by the above methods. The particle swarm optimization (PSO)^16^, artificial fish school algorithm (AFSA)^17^ and Cuckoo Search (CS)^18^ are used for comparison. The population size and iteration numbers of these intelligent algorithms are the same to ensure rigorous comparison. The search paths and results are shown in Fig. 2.

The detailed values are shown in Table 1. The comparison of the tracking trajectory and the optimization results show that BA has advantage of high convergence speed and computational accuracy because the gradient of the optimization deviation is the largest and the optimization results are closest to the true value.

**Table 1:** Results analysis of different methods.

Algorithm	Search results	Optimal solution	Time running
PSO	[0.0644, 0.0518]	0.4025	0.484925
AFSA	[0.0011, 0.0052]	0.01583	0.585477
CS	[0.0083, 0.0198]	0.06764	0.254225
BA	[0.0023, − 0.0014]	0.007828	0.154801

The ideal search results are [0, 0] and ideal solution is 0.

## Methods

### BatOMP sparse decomposition

#### General atomics designed for typical signals

**Figure 3:**
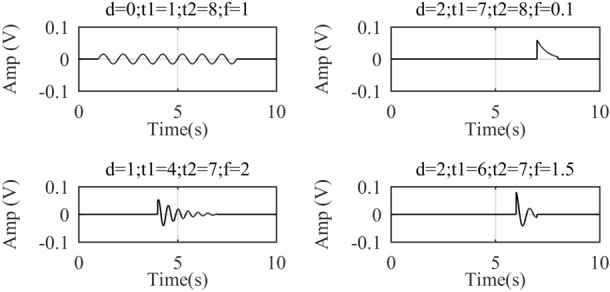
Time domain waveforms of g-atoms with different parameters.

For sinusoidal-like and one-sided decaying oscillatory signals, *g*-atoms are constructed:

12 $$ g\left( {c,d,t_{1} ,t_{2} ,\tau ,f,\varphi } \right) = \left\{ {\begin{array}{*{20}l} {ce^{{\left( { - d\left( {t - t_{1} } \right)} \right)}} \cos \left( {2\pi f\left( {t - t_{1} } \right) + \phi } \right),} \hfill & {t \in \left[ {t_{1} ,t_{2} } \right]} \hfill \\ {0,} \hfill & {others} \hfill \\ \end{array} } \right., $$

where *c* is the normalization factor to ensure that the original signal has the same energy as its sparse decomposition results; *d*: the attenuation factor; *t*: the sampling time; *t*_1_: start point of atomic appearance; *t*_*2*_: the ending point; *f*: signal frequency and *ϕ*: phase. The time domain waveforms of *g*-atoms with different parameters are shown in Fig. 3.

When the attenuation factor *d* = 0, the *g*-atom degenerates to standard sine wave; when *d* increases, the *g*-atom performs sinusoidal damped oscillation. Therefore, this atom has a strong match with sinusoidal signals, and single-sided oscillatory decay signals.

For kind of triangle waves, charge–discharge waves, and bilateral decay oscillation signals, *t*_*r*_ atoms are constructed:

13 $$ t_{r} \left( {c,d_{1} ,{\text{d}}_{{2}} {\text{,t}}_{{0}} {,}t_{1} {,}t_{2} {\text{,f,}}\varphi {,}\alpha } \right) = \left\{ {\begin{array}{*{20}l} {c \times e^{{\left( { - d_{1} *\left( {t_{1} - t} \right)} \right)}} \times \cos \left( {2\pi f\left( {t_{1} - t} \right) + \phi } \right),} \hfill & {t \in \left[ {t_{0} ,t_{1} } \right]} \hfill \\ {0,} \hfill & {others} \hfill \\ {\eta \times c \times e^{{\left( { - d_{2} *\left( {t - t_{1} } \right)} \right)}} \times \cos \left( {2\pi f\left( {t - t_{1} } \right) + \phi } \right),} \hfill & {t \in \left[ {t_{1} ,t_{2} } \right]} \hfill \\ \end{array} } \right., $$

where *d*_1_ and *d*_2_ are the bilateral damping factors; *t*_*0*_ is the bilateral boundary of the atomic; [*t*_1_, *t*_2_] is the atomic time range; and *η* is the bilateral scaling factor.

**Figure 4:**
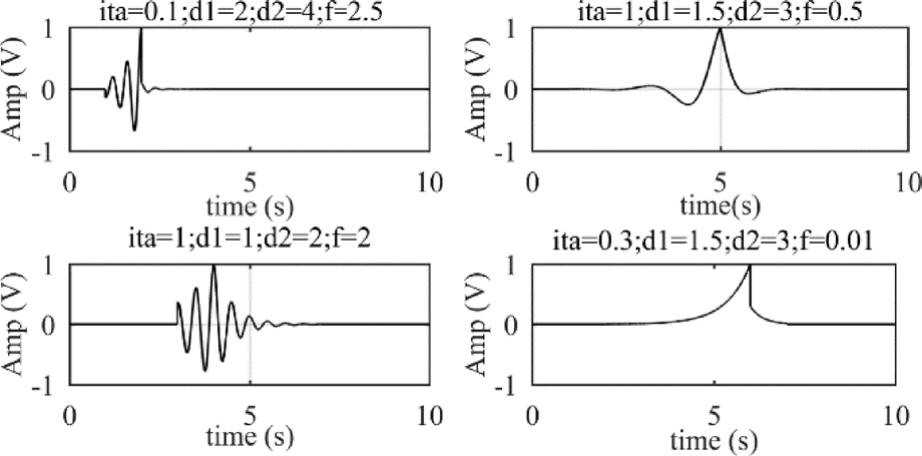
Time domain waveforms of *t*_*r*_-atoms with different parameters. ‘ita’ represents *η.*

The time domain waveforms of *t*_*r*_-atoms with different parameters are shown in Fig. 4. When the bilateral scaling factor *η* = 0, the *t*_*r*_-atom degenerates to single-sided oscillating atom (reverse-order *g*-atom); when 0 < *η* < 1 and the atomic frequency is low enough, the *t*_*r*_-atom behaves as a charge–discharge triangle wave; when *η* = 1, the *t*_*r*_-atom with low-frequency behaves as a triangle-like wave, and behaves as bilateral oscillating decay signal with high-frequency.

The above analysis shows that the constructed *g*-atoms and *t*_*r*_-atoms are very flexible and could match almost typical testing signals by parameter adjustment.

#### Dictionary training algorithm

A dictionary training algorithm based on feature parameters was studied to determine the key parameters of feature base-atom and to balance the completeness and redundancy for redundant dictionary library construction.

When constructing the redundancy dictionary, we firstly use the short-time Fourier transform to initially determine the frequency *f*_*s*_ and phase *ϕ*_*s*_ of the target signal in the original data *x*. Then, standard sine atom *s*_*0*_ = sin(2*πf*_*s*_*t* + *ϕ*_*s*_) is constructed. The correlation detection technique is used by calculating the correlation function of the original data and the sine atom, and the upper and lower envelopes of the correlation function are obtained by searching the positive and negative peaks of the correlation function. The points of the maximum positive gradient between the positive and negative peaks are the extracted range of the target signal. Thus, the signal sample *E*_*i*_ in the original observation sequence is extracted. The time information section obtained by the above calculation determines the time domain parameters such as *t*_*0*_, *t*_*1*_ and *t*_*2*_ of the characteristic atom.

Accurate time and frequency domain parameters are obtained by Hilbert transform of *E*_*i*_.

14 $$ R\left( t \right) = E_{i} \left( t \right) \times h\left( t \right), $$

where: *h(t)* is the Hitch transform factor.

Complex analytic signal as follows is constructed:

15 $$ z\left( t \right) = x\left( t \right) + iR\left( t \right) = Ae^{i\phi \left( t \right)} , $$

where, *A*(*t*) is the amplitude function:

16 $$ A\left( t \right) = \sqrt {E_{i}^{2} \left( t \right) + R^{2} \left( t \right)} , $$

and, *ϕ*(*t*) is the phase function:

17 $$ \phi \left( t \right) = \arctan \frac{R\left( t \right)}{{E_{i} (t)}}. $$

The instantaneous frequency of *E*_*i*_ is given by (18):

18 $$ f\left( t \right) = \frac{d\phi (t)}{{dt}}. $$

The base-atom is obtained with the time information gained by the correlation detection and localization algorithm and the time–frequency parameter information obtained by Hilbert transform as the reference. And the redundant dictionary of this feature atom is constructed by performing equal-step discrete expansion of the time–frequency parameters on both sides of the reference values.

19 $$ {\text{D = }}\left\{ {\begin{array}{*{20}c} {g_{1}^{1} ,\,g_{2}^{1} ,\, \ldots g_{M}^{1} } \\ {g_{1}^{2} ,\,g_{2}^{2} ,\, \ldots g_{M}^{2} } \\ \vdots \\ {g_{1}^{N} ,\,g_{2}^{N} ,\, \ldots g_{M}^{N} } \\ \end{array} } \right\}, $$

where, *D*(:, *j*) = {*g*_*j*_^*i*^|*i* = 1,2,…,*N*}, denoting the set of atoms consisting of extensions of the characteristic base-atom *g*_*i*_^*0*^. Atoms has the same length *N* as *x*.

### BatOMP improved sparse decomposition algorithm

The optimization-seeking process can be viewed as a global optimization problem. In order to solve the problems of large computation and low efficiency of existing matching tracking algorithms, the adaptive matching tracking algorithm, BatOMP, with fast convergence and accurate approximation is studied by combining BA into the OMP algorithm.

*For* BatOMP, the bat individual positions *P*_*i*_ represent the atoms column index in the redundant dictionary *D*, thus: *g*_*i*_ = *D*(*:,P*_*i*_). And for noise-containing signal extraction, the fitness function of the traditional sparse decomposition is improved to take the ratio of the *ℓ*-2 norm of the residual and the inner product as the fitness function. The target signal tends to be regular signals and most random noises obeys Gaussian distributions with zero mean error. So, the *ℓ*-2 norm of the former is greater than the latter. In consequence, the smaller the fitness indicates that the residual sequence contains smaller effective signal components and higher signal-to-noise separation. In addition, the larger the inner product, the better matchs between the atom and the residual. So, the optimal individual bat position *P*_*b*_ is determined and saved according to (20).

20 $$ Fit^{n} \left( {P_{best}^{n} } \right) = Fit_{n} \left( {g_{best}^{n} } \right){\text{ = arg }}\mathop {{\text{min}}}\limits_{{N_{pop} }} \left( {\frac{{\left\| {\xi_{i}^{n + 1} } \right\|_{2}^{{}} }}{{\left\langle {\xi_{i} \cdot g_{i} } \right\rangle }}} \right), $$

and

21 $$ \xi_{i}^{n + 1} = \xi_{i}^{n} - A^{T} \left( {A^{T} \times A} \right)^{ - 1} \times A^{T} *\xi_{i}^{n} ,A = \left[ {g_{1} ,g_{2} , \ldots ,g_{n} } \right], $$

where *A* is the matched dictionary, composed by the selected best matching atoms.

**Figure 5:**
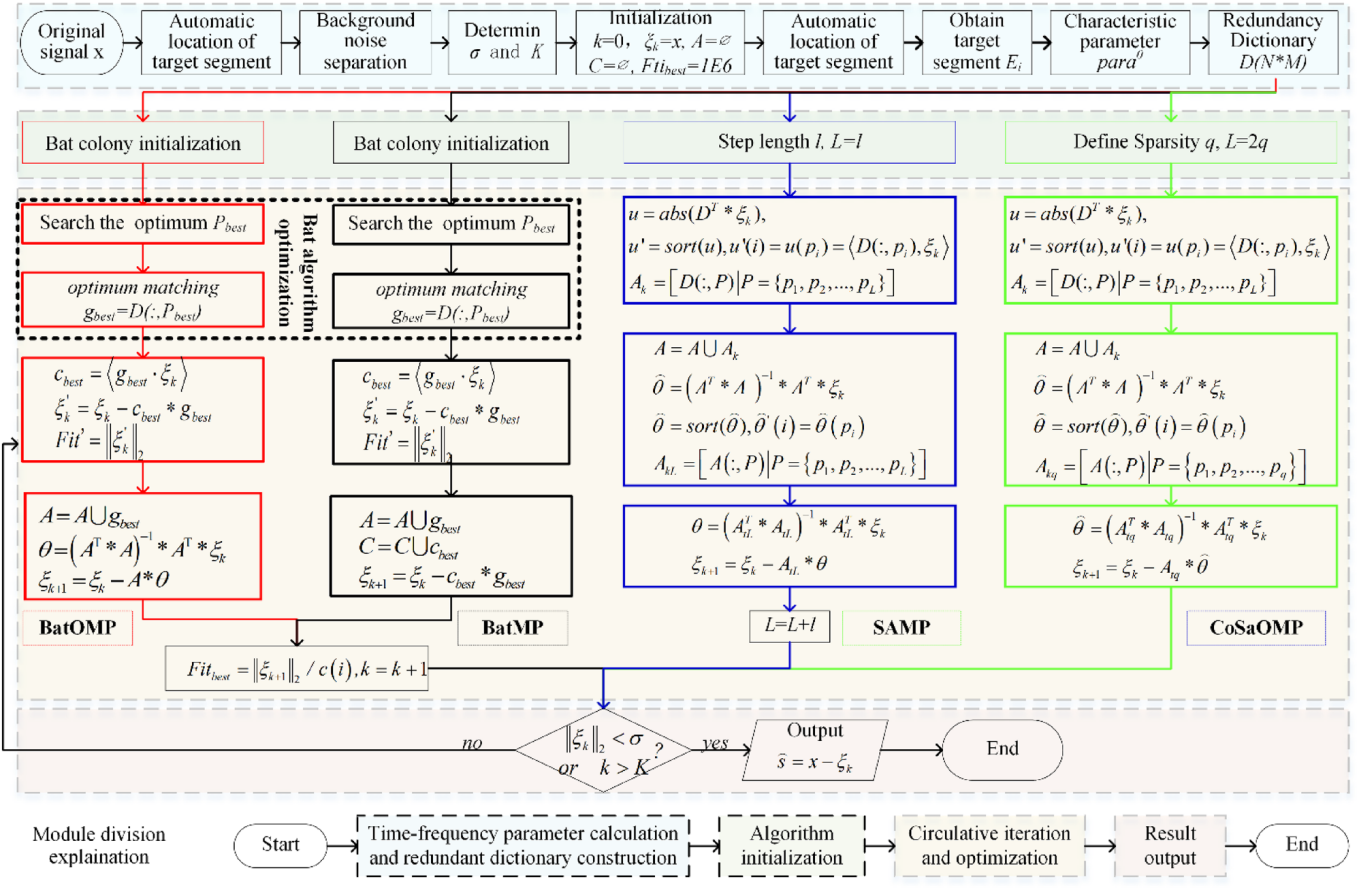
The overall flow chart of signal extraction algorithms.

The flow chart of BatOMP is as follows:
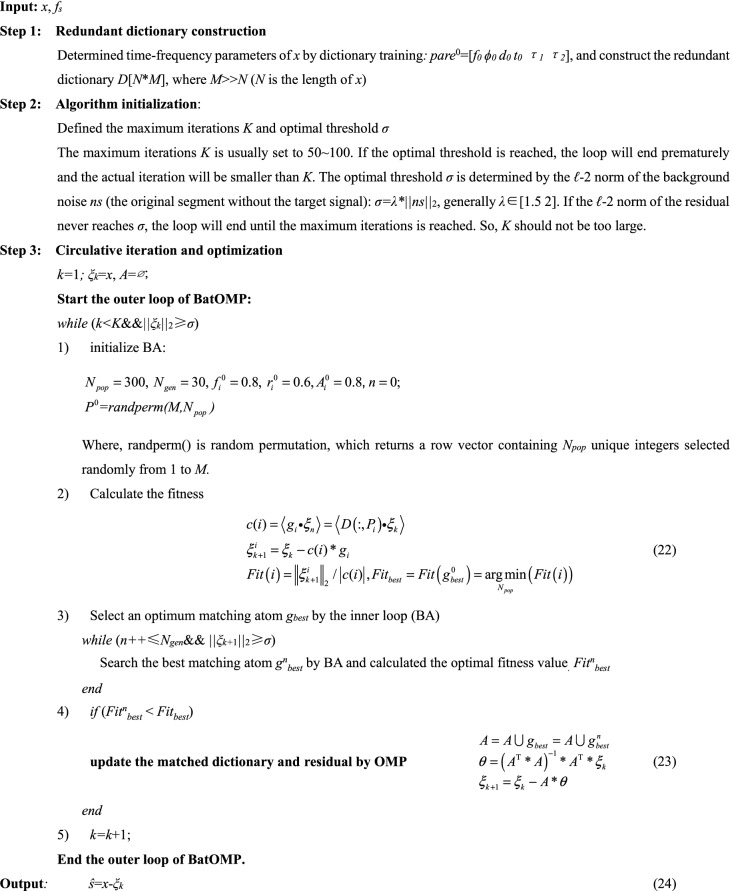


The overall flow chart of the proposed signal extraction algorithm is shown in Fig. 5. The flow of sparse decomposition based on BA and MP (BatMP), CoSaMP and SAMP are also presented for comparison. Different algorithms are distinguished by different border colors. All of the four methods consist of four main modules: 1. redundant dictionary construction, 2. algorithm initialization, 3. circulative iteration and optimization, and 4. result output. In this paper, module 1 and 4 are almost same for the different methods, module 2 is slightly different, and the differences are mainly reflected in module 3.

### Experiments

We constructed a nonstationary signal *x* to test the methods described above:

25 $$ x = ns + s = \left\{ {\begin{array}{*{20}l} {ns + s_{1} ,} \hfill & {t_{1s} < t < t_{1e} } \hfill \\ {ns + s_{2} ,} \hfill & {t_{2s} < t < t_{2e} } \hfill \\ {ns,} \hfill & {otherwise} \hfill \\ \end{array} } \right. = \left\{ {\begin{array}{*{20}l} {wgn + 20 \times {\text{exp}}\left( { - \frac{{{\text{5e}} - {4}}}{{\sqrt {{(1} - {0}{\text{.0005}}^{{2}} {)}} }} \times {(2}\pi \times {90} \times {\text{(t}} - {0}{\text{.156))}}^{{2}} } \right) \times {\text{cos(2}}\pi \times {90} \times {\text{(t}} - {0}{\text{.156))}},0 < t < 1} \hfill & {0 < t < 1} \hfill \\ {wgn + 180 \times \left\| {{\text{exp(}} - {15} \times {\text{t)}} \times {\text{sin(2}}\pi \times {30} \times {\text{t + }}\pi {/2)}} \right\|,} \hfill & {0.5 < t < 0.67} \hfill \\ {wgn,} \hfill & {otherwise} \hfill \\ \end{array} } \right., $$

where *s* is nonstationary target signal including pulse signal *s*_1_ and partial discharge signal *s*_2_ distributed in different regions, and *ns* is background noise subjecting to Gauss distribution. The sampling rate *f*_*s*_ = 1500 Hz, sampling time *T* = 1 s, and SNR is 7.402 dB. Thus, the sequence length *N* is 1500.

The PC for the testing features a i7-8550U CPU Core(TM) @ 1.80 GHz with 16.0 GB RAM, 4 cores and 8 Logic processors, running the 64 bit operating system.

### Time–frequency parameter calculation

**Figure 6:**
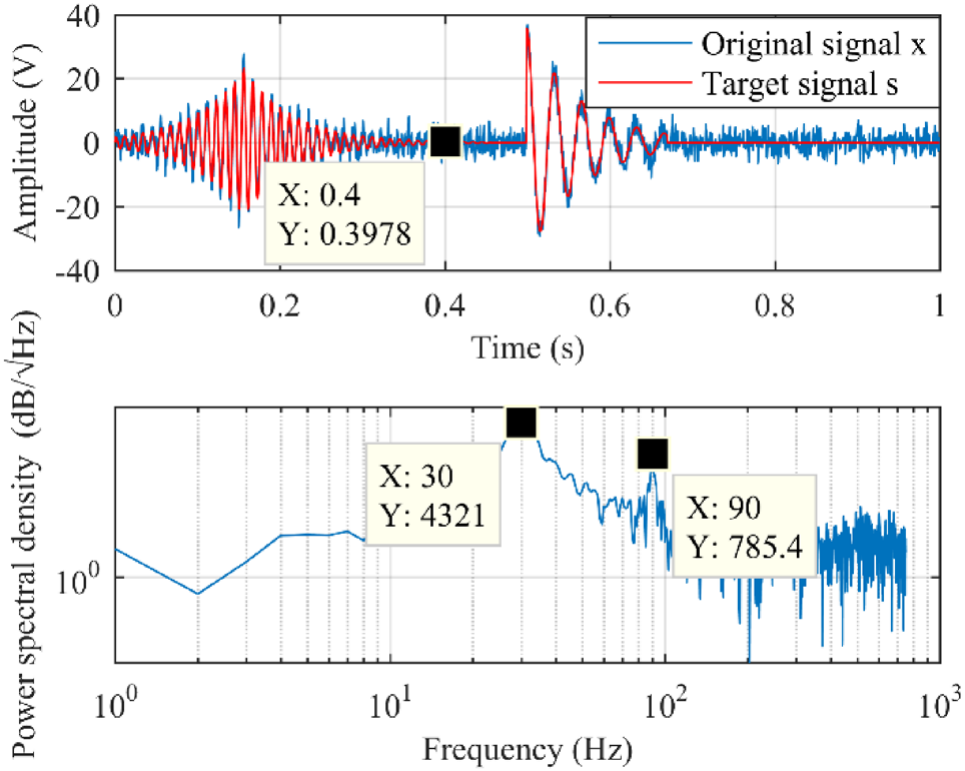
Waveform of the testing signal. The upper and lower subplot denote the time domain and frequency domain waveforms, respectively; red and blue lines in the upper subplot denote the original signal *x* and the target signal *s* = *s*_1_ + *s*_2_, respectively.

First, time and frequency analysis was performed, and results were shown in Fig. 6. From Fig. 6, after 0.4 s, *s*_1_ decreases to zeros with the action of attenuation term.

**Figure 7:**
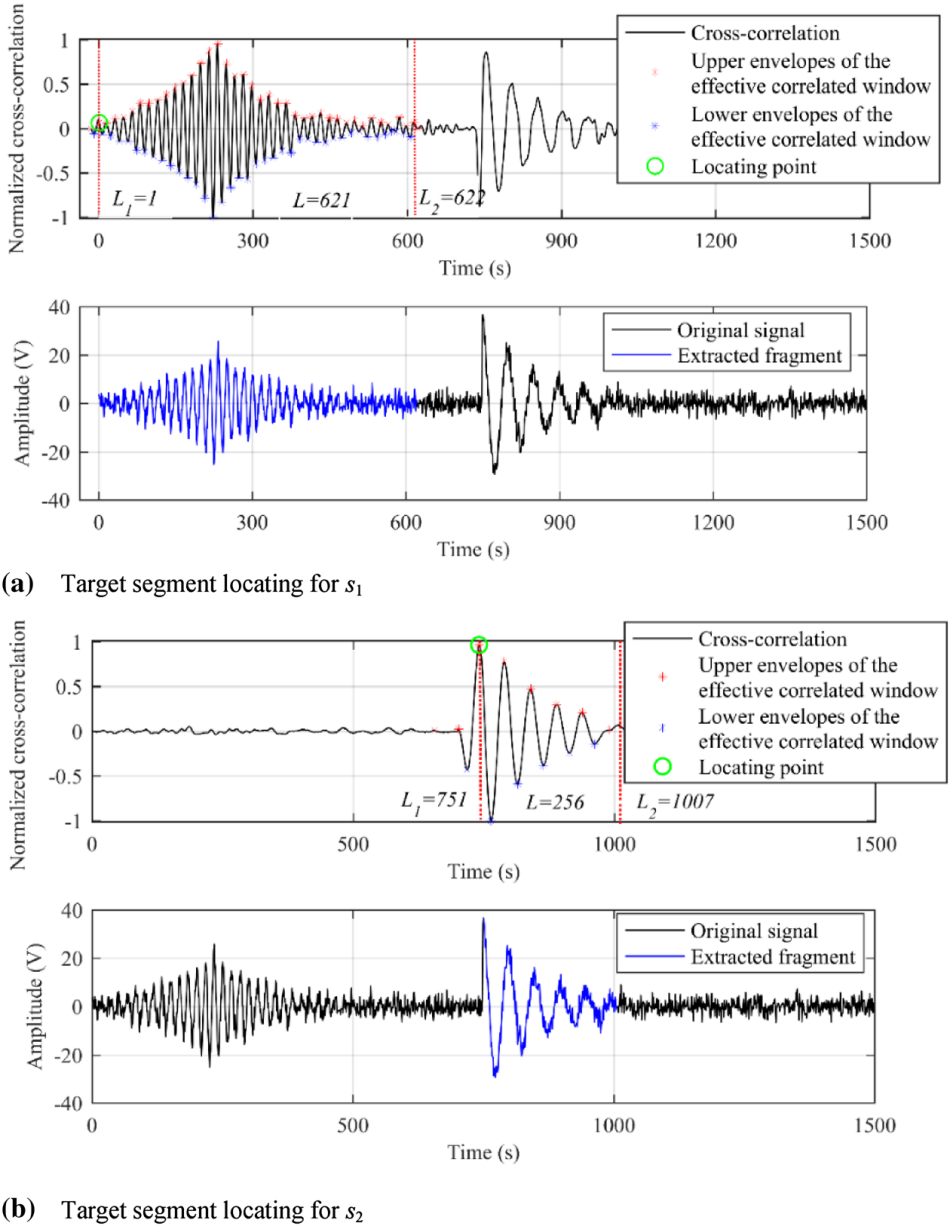
Target segment extraction based on correlation detection technique. The upper and lower subplot denoted the segmentation result of signal components *s*_1_ and *s*_2_, respectively. In the upper subplots, the black solid lines are cross-correlation functions; red and blue scatters describe the envelopes of the effective correlated windows; and red dotted line are calculated time range. The green cycles indicate the start–end of the target segments.

Secondly, time information of the target signal was calculated by the correlation detection and localization algorithm described above, shown in Fig. 7. *L*_1_ and *L*_*2*_ are the calculated start and end indexes of the target segments, and *L* is the length of the segmentations. Accordingly, *t*_1_ = *L*_1_/fs ≈ 0 s, *t*_2_ = *L*_2_/fs ≈ 0.415 s for *ŝ*_1_, and *t*_1_ = *L*_1_/fs ≈ 0.501 s, *t*_2_ = *L*_2_/fs ≈ 0.671 s for *ŝ*_2_.

### Different dictionaries construction and testing

After determining the key parameters of *s*, the redundant dictionary GT consisting of the two new atoms was created by dictionary training algorithm. And two typical redundant dictionaries were built by Discrete Cosine Transformation (DCT) and Gabor dictionary for comparison. The dictionaries’ sizes are shown in Table 2.

**Table 2:** The dictionary sizes.

Dictionary	Size
Gabor	1500 × 863,952
DCT	1444 × 1,048,576
GT	1500 × 593,190

When built the DCT dictionary, perfect square was required for the column length, so the nearest perfect square 1444 around *N* was selected.

**Figure 8:**
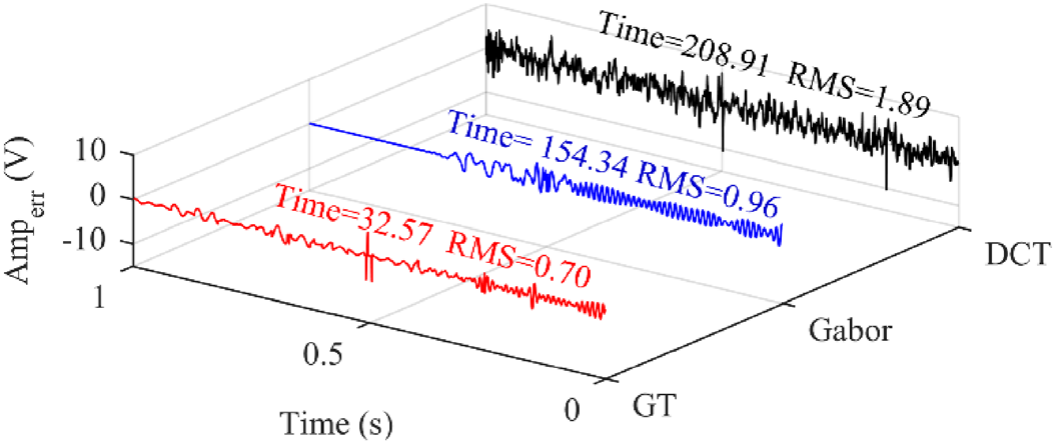
Dictionary testing.

Since the OMP method will iterate over the whole dictionary repeatedly, the extraction results are relatively accurate. In view of this, we take the results of OMP from statistical analyses to illustrate the performance of the different dictionaries, as shown in Fig. 8. The vertical axis represents the deviation between the extracted signal *ŝ* and actual signal *s*:

26 $$ Amp_{err} = s - \widehat{s}. $$

We quantified the errors by RMS, the root mean square value of *Amp*_*err*_. The time–frequency parameters were not considered when building the DCT dictionary by MATLAB, so there was the maximum deviation in the correlative results. The atom expression of Gabor dictionary is as follows:

27 $$ gabor(i) = \frac{1}{\sqrt a } \times \exp \left( { - \pi \times \left( {\frac{i - \Delta t}{z}} \right)^{2} \times \cos (2\pi f(i - \Delta t) + \phi )} \right). $$

We can see the lack of unilateral oscillation atoms by contrast with *g*-atom and *t*_*r*_-atom. Relatively, GT dictionary is completer and more accurate, so OMP based on GT dictionary gave minimum errors and the shortest optimizing time.

### Algorithm performance testing

The algorithms involved in the article including MP, OMP, SAMP, CoSaMP, BatMP and the proposed BatOMP were carried out for performance comparison.

**Figure 9:**
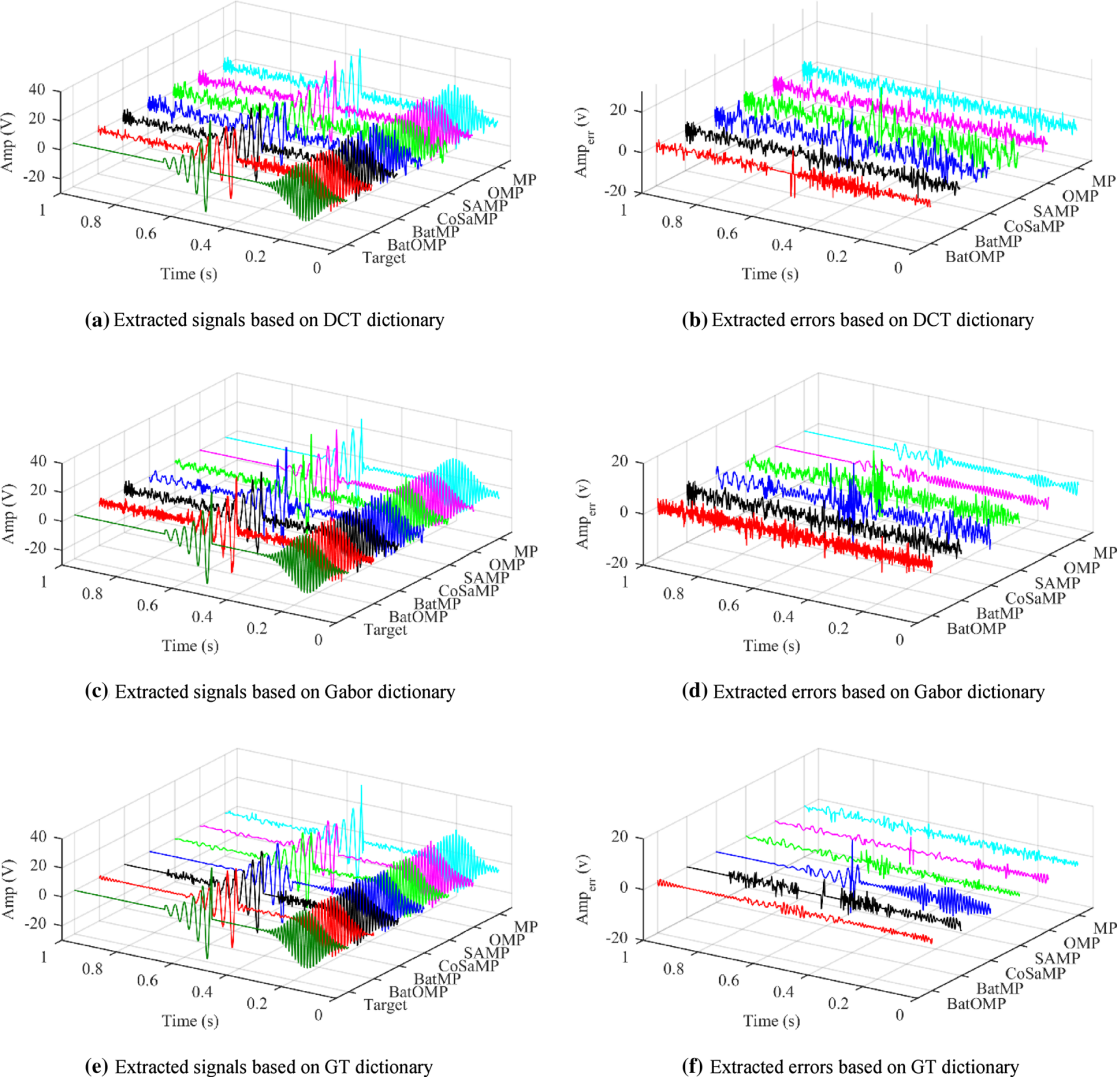
Signal extraction results. Colors: jasper: the target signal *s*; red: result of BatOMP; black: result of BatMP; bule: result of CoSaMP; green: result of SAMP; pink: result of OMP; skyblue: result of MP.

The extraction results and corresponding errors obtained by different methods were shown in Fig. 9. Figure 9a,c,e display the extracted signals *ŝ* by different methods based on DCT, Gabor and GT dictionary respectively. And Fig. 9b,d,f presente corresponding errors obtained by Eq. (26).

**Figure 10:**
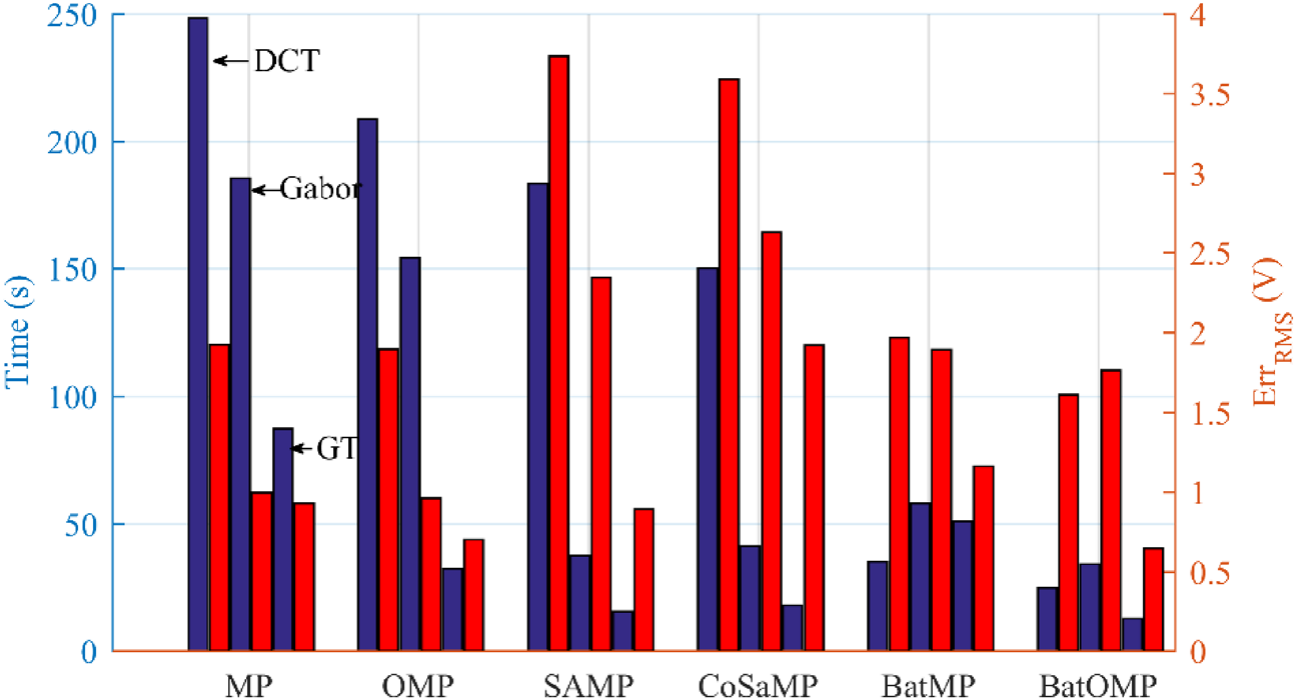
Quantitative analysis of experimental results. Blue bares mark the running time (the left axis) and red bars indicate the extraction errors (the right axis). For each method, the three bars of a sort from left to right represent the result of DCT, Gabor and GT dictionary respectively.

The efficiency analysis of different algorithms and dictionaries are shown in Fig. 10 and Table 3.

**Table 3:** Quantitative analysis of experimental results.

Algorithm	Elapsed time (s)			Square error (V)			CPU occupancy rate (%)
	DCT	Gabor	GT	DCT	Gabor	GT	
MP	248.45	185.62	87.38	1.92	1.00	0.93	58
OMP	208.91	154.34	32.57	1.89	0.96	0.70	57
SAMP	183.6	37.70	15.51	3.74	2.34	0.89	70
CoSaMP	150.11	41.41	18.01	3.59	2.63	1.92	55
BatMP	35.26	58.19	51.16	1.97	1.89	1.16	26
BatOMP	25.09	34.35	12.77	1.61	1.77	0.65	26

Because that the MP and OMP method traversed through the whole dictionary, the accuracies are relatively high and the latter is superior to the former.

The step length *l* of SAMP and sparsity *q* of CoSaMP were determined by expert experience. Because a certain amount atoms have to choose every time, there are clearly overextraction for these two methods. It’s important to note that better parameters may be obtained by trial and error, but it is not suitable for real-time data processing.

**Figure 11:**
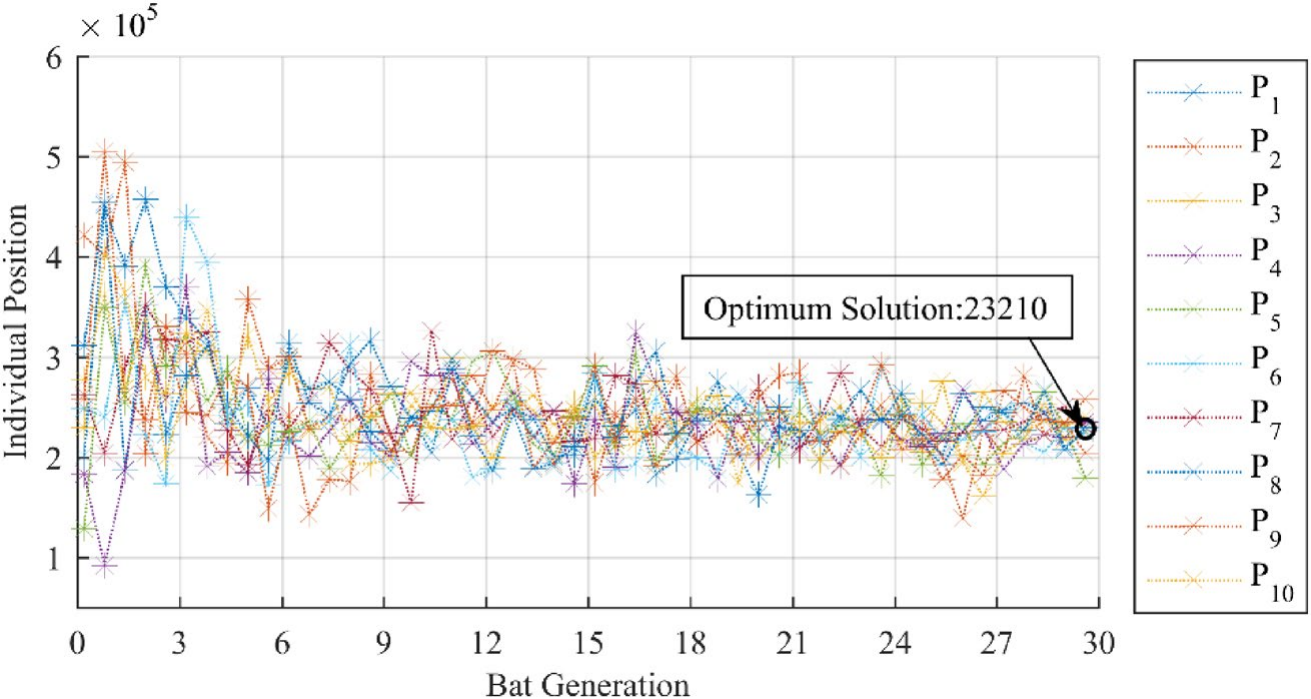
A complete search process of the bat colony. Obviously, the bats gradually converge to the best solution from the original scattered position.

For BatOMP, the best match atoms are determined by bat colony optimization. Every time before the searching, the bat individuals randomly scattered over the whole dictionary, and then gradually gather to the optimum solution through local optimization and global optimization. The optimal trajectories of ten bat individuals were randomly selected to show the convergence process, as shown in Fig. 11. The optimum solution is the index of the optimum matching atom in the redundant dictionary.

The difference between BatMP and BatOMP is similar to MP and OMP. BatOMP based on GT dictionary occupied the highest precision, probably because that MP and OMP took the inner product as the fitness function which leading to suboptimum for signal extraction. So, the results reflect the availability of the new fitness in some extent.

Moreover, the first four methods executed vast and complex matrix computations many times during the optimizing period, so they are time-consuming and require very high CPU occupancy rate compared with BatOMP. In other words, BatOMP can be widely used even on low lever machines. This is important for the occasions without algorithmic workstation and high-performance computer, i.e. field data processing or low cost testing.

## Conclusion

For nonstationary signal extraction, the dictionary training algorithm based on feature parameters is firstly used to determine the key parameter range of feature atoms, which can effectively reduce the redundancy while ensuring the completeness of the redundant dictionary; the bat algorithm combined with OMP is proposed to transform the signal sparse decomposition problem into an optimization problem with ratio of the *ℓ*-2 norm of the residual and the inner product as the fitness, which can improve the efficiency of the sparse decomposition algorithm. The experimental results showed that compared with other methods, the BatOMP algorithm is occupied with high efficiency, which can extract nonstationary signals form noise background without over constrained prior knowledges and avoid excessive decomposition. Testing results show that the proposed algorithm outperforms previous method in speeding up the convergence procedure and meanwhile ensuring high accuracy. Compared with the existing sparse decomposition algorithm, BatOMP requires much lower levels of hardware configuration. So, the new method will be helpful for to reducing data processing cost and enlarging the application fields.
